# Characterization of the Dioscorin Gene Family in *Dioscorea alata* Reveals a Role in Tuber Development and Environmental Response

**DOI:** 10.3390/ijms18071579

**Published:** 2017-07-20

**Authors:** Linya Liu, Yacheng Huang, Xiaolong Huang, Jianghua Yang, Wenqiang Wu, Yun Xu, Ziwen Cong, Jun Xie, Wei Xia, Dongyi Huang

**Affiliations:** 1 Institute of Tropical Agriculture and Forestry, Hainan University, Haikou 570228, China; liulinya913@126.com (L.L.); yachenghuang1314@126.com (Y.H.); wuwenqiang1314@126.com (W.W.); xuyun2000513@126.com (Y.X.); wencycong@gmail.com (Z.C.); junxie96@126.com (J.X.); saizjxiawei@hainu.edu.cn (W.X.); 2 School of Biological Sciences and Technology, Liupanshui Normal University, Liupanshui 553001, China; 3 Hainan Key Laboratory for Sustainable Utilization of Tropical Bioresources, Institute of Tropical Agriculture and Forestry, Hainan University, Haikou 570228, China; huangxialong@hainu.edu.cn; 4 Rubber Research Institute, Chinese Academy of Tropical Agricultural Sciences, Danzhou 571737, China; jianghuayang@163.com

**Keywords:** dioscorin, cloning and expression, tuber development, promoter, *Dioscorea alata* L.

## Abstract

Dioscorin is one of the major soluble proteins in yam tubers. Unlike other well-known plant storage proteins, such as patatin and sporamin, dioscorin is argued for its function as storage proteins, and the molecular mechanisms underlying its expressional complexity are little understood. In this study, we isolated five dioscorin genes from *Dioscorea alata* L., comprising three class A (*Da-dio1*, -*3* and -*4*) and two class B (*Da-dio2* and -*5*) isoforms. Expressions of all dioscorin genes gradually decreased in mother tubers during yam sprouting and regrowth. On the other hand, all dioscorin genes accumulated transcripts progressively with tuber development in new tubers, with *Da-dio5* being the most prominent isoform. In yam leaves, the expressions of *Da-dio*5 were up-regulated by the treatments of five phytohormones (gibberellic acid, salicylic acid, indole-3-acetic acid, abscisic acid, and ethylene), and three abiotic stresses (high-temperature, low-temperature and drought). To further elucidate the regulatory mechanisms of *Da-dio5* expressions, transgenic *Arabidopsis* plants harboring the *Da-dio5* promoter-β-glucuronidase (GUS) fusion were generated. GUS staining showed that expressions of the *Da-dio5* promoter were detected mainly in the shoot apical meristem (SAM) and hypocotyls, and enhanced by the treatments of the five hormones, and the three abiotic stresses mentioned above. These results suggest diverse roles of *Da-dio5* in yam sprouting, regrowth, and tuberization, as well as in response to enviromental cues.

## 1. Introduction

Yams (*Dioscorea* spp.) are members of the monocotyledonous family *Dioscoreaceae*. More than 600 yam species have been cultivated worldwide [1], consumed as an important source of food in some African and Asian countries due to the high contents of carbohydrate and nutritionally relevant proteins (1–3%) in fresh tubers [2,3]. In China, yams are traditionally used as medicinal foods to strengthen the spleen, kidney, liver, and stomach; to reduce phlegm and fatigue; and to cure chronic diarrhea and diabetes [4]. The beneficial properties of yams have recently been attributed to the storage protein dioscorin [5,6,7,8]. Consequently, the functional properties and pharmaceutical potential of dioscorin have attracted increasing attention [9,10].

Dioscorin is the major protein in yam tubers, accounting for approximately 80–85% of the total soluble proteins [11]. A previous study revealed that the major tuber proteins in yam tubers are encoded by members of gene family classes of A and B [1]. The protein sequences of the members from these two classes are 67–75% identical in *Dioscorea cayenensis* [12]. However, dioscorin has been argued for its function as a storage protein despite its high abundance in yam tubers. Unlike most other storage proteins, dioscorin also has types of enzymatic activities, such as trypsin inhibitor (TI), carbonic anhydrase (CA), dehydroascorbate (DHA) reductase, monodehydroascorbate (MDA) reductase activities, and unique lectin activities [13,14,15,16]. Moreover, dioscorin possesses antioxidant properties, with an ability to scavenge both 1,1-diphenyl-2-picrylhydrazl radicals and hydroxyl radicals [17]. Dioscorin was even found to inhibit angiotensin-converting enzyme activity [18]. The well-known tuber storage proteins, such as sporamin from sweet potato and patatin from potato, also contribute to pest and pathogen resistance, as well as resistance to abiotic stresses [1,17,19,20]. These findings suggest that the so-called storage proteins including dioscorin may have dual roles related to storage and defense in plants.

Although the predominant protein in yam tubers, compared to patatin and sporamin, dioscorin has been less intensively studied. In the present study, we isolated five dioscorin genes (i.e., *Da-dio1*–*5*) in a cultivar of *D. alata* L. and explored their expression characteristics in relation to yam sprouting, regrowth, and tuberization, as well as in response to the treatments of hormones and abiotic stresses. Furthermore, the promoter-β-glucuronidase (GUS) reporter technique was exploited in transgenic *Arabidopsis* plants to investigate the molecular mechanisms underlying the complex expressional control for *Da-dio5*, a major *Da-dio* isoform. It is expected that our results reported here will help understand the roles of dioscorin in yam tubers, and provide useful information for understanding its transcriptional regulations.

## 2. Results

### 2.1. Identification of the Da-dio Gene Family

**Figure 1:**
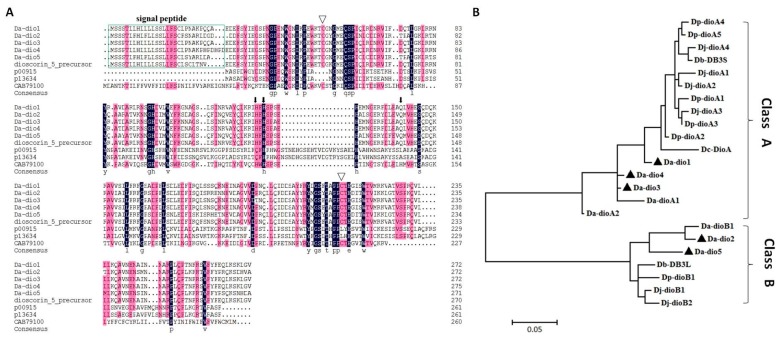
Amino acid sequence alignment and phylogeny of the five *Da-dio* genes. (**A**) Alignment of the sequences of five Da-dio proteins this study, a *D. japonica* dioscorin homolog (dioscorin-5 precursor), and three carbonic anhydrases (CAs) from human (P00915), mouse (P13634) and *Arabidopsis* (CAB79100), respectively. Putative residues related to CA activity are indicated with a black arrow. Two cysteine residues that are implicated in activities of trypsin inhibitor (TI), dehydroascorbate (DHA) reductase, and monodehydroascorbate (MDA) reductase are indicated with an inverted triangles; Identical or conserved amino acids are shaded in black or red, respectively; (**B**) phylogenetic tree constructed for 23 dioscorin proteins from different yam species using MEGA version 6.0 (www. megasoftware.net). The five dioscorin proteins identified in this study are indicated with black triangles. The other dioscorin proteins are as follows: three *D. alata* dioscorins (*Da-dioA1*, AF242551; *Da-dioA2*, AF245019; *Da-dioB1*, AF243526), six *D. japonica* dioscorins (*Dj-dioA1*, AM849818; *Dj-dioA2*, AM849819; *Dj-dioA3*, AM849820; *Dj-dioA4*, AM849821; *Dj-dioB1*, AM849816; *Dj-dioB2*, AM849817), six *D. pseudojaponica* dioscorins (*Dp-dioA1*, GQ246171; *Dp-dioA2*, GQ246172; *Dp-dioA3*, GQ246173; *Dp-dioA4*, GQ246174; *Dp-dioA5*, GQ246175; *Dp-dioB1*, GQ246170), two *D. batatas* dioscorins (*Db-DB3S*, AB178473; *Db-DB3L*, AB178472), and one *D. cayenensis* dioscorin (*Dc-dioA*, X76187).

The full-length cDNAs of five *Da-dio* genes were cloned and submitted to GeneBank (*Da-dio1*, KX237676; *Da-dio2*, KX237677; *Da-dio3*, KX237678; *Da-dio4*, KX237676; *Da-dio5*, KX237676). The open reading frame (ORF) of the five *Da-dio* genes were 819–831 base pair (bp) long, and predicted proteins of 272–276 amino acids (aa), molecular weights (MW) of ~30 kDa, and theoretical isoelectric point (pI) values of 6.17 to 7.02 (Table 1). Multiple sequence alignments were performed upon the five Da-dio proteins identified here, a dioscorin homolog (dioscorin-5 precursor) from *Dioscorea japonica* and three carbonic anhydrases (CAs) from human (P00915), mouse (P13634) and *Arabidopsis* (CAB79100), respectively. As shown in Figure 1A, the five Da-dios contained three putative residues that influence CA activity, and two cysteine residues that relate to activities of TI, DHA reductase, and MDA reductase. In addition, all dioscorin proteins contained a putative signal peptide (amino acids 23–25). A phylogenetic tree (Figure 1B) was constructed for the five Da-dios and 18 NCBI-downloaded dioscorin sequences of five *Dioscorea* species (*D. alata*, *D. japonica*, *D. pseudojaponica*, *D. batatas* and *D. cayenensis*). The five Da-dios identified this study were grouped clearly into class A (*Da-dio1*, *Da-dio3* and *Da-dio4*) and class B (*Da-dio2* and *Da-dio5*). Amino acid sequence alignments showed a high similarity among the Da-dios of different cultivars, being 91–97% identical within the same subfamily, and 68% similar across the subfamilies (Table S1 and Figure 1B).

### 2.2. Expression of the Five Da-dios During Tuber Development

**Figure 2:**
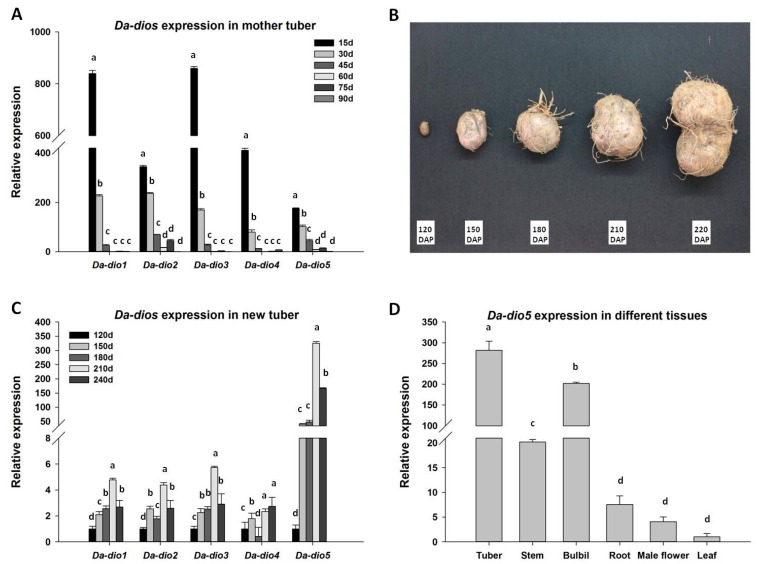
Quantitative real-time RT-PCR (qRT-PCR) analysis of expression levels of five *Da-dio* genes during tuber development in *D. alata* cv. Hainan No. 56. (**A**) Expression changes of five *Da-dio* genes in mother tubers during the vine growth stage; (**B**) the picture of new tubers formed at different days after planting (DAP). Five stages of new tuber development are tuber formation (120 DAP), rapidly bulking I (150 DAP), rapidly bulking II (180 DAP), maturing (210 DAP), and harvesting (240 DAP); (**C**) Expression changes of five *Da-dio* genes during new tuber development; (**D**) Expression of *Da-dio5* in six *D. alata* tissues, viz. tuber, stem, bulbil, root, male flower and leaf. Values are presented as the mean ± standard error of three independent biological replicates. Different letters indicate significant differences (*p* < 0.05) according to one-way analysis of variance.

*D. alata* L. propagates mainly via tubers, the modified stem. Tuber development is accompanied by a variety of biochemical and morphological changes. To explore the roles of the five *Da-dio* genes during yam sprouting and regrowth, we examined their expressions in the mother tubers at 15, 30, 45, 60, 75 and 90 days after planting (DAP). All five *Da-dio* genes were expressed at highest abundance in mother tubers at 15 DAP. As the vines continued to grow, the expression of the five *Da-dio* genes gradually decreased until reaching almost undetectable levels at 90 DAP (Figure 2A). To further investigate whether the dioscorin gene expressions are associated with tuber development, the *Da-dio* transcripts were investigated in new tubers at 120, 150, 180, 210 and 240 DAP, corresponding to different stages of tuber development (Figure 2B). As shown in Figure 2C, the expressions of five *Da-dio* genes increased in a similar pattern in the new tubers except for a difference in the fold-change. The expressions of *Da-dio1*–*4* were up-regulated by 3–5-fold, whereas almost 300-fold was observed for *Da-dio5* at the beginning of the maturation stage (210 DAP). These results suggested that expressions of the five dioscorin genes are closely correlated with yam sprouting, regrowth, and tuber development, of which *Da-dio5* seems to be the most important member implicated in tuber formation and development.

### 2.3. Expression Pattern and Localization of Da-dio5

**Figure 3:**
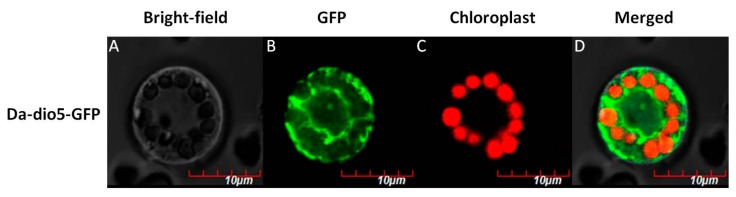
Subcellular localization of Da-dio5–GFP by transient expression in rice protoplasts: (**A**) bright field image; (**B**) transient expression of GFP; (**C**) chloroplast autofluorescence; and (**D**) merged GFP and chloroplast image. Scale bar = 10 µm.

To further understand the functions of *Da-dio5*, its expressions were examined in six *D. alata* tissues, viz. tubers, stems, roots, bulbils, male flowers and leaves. *Da-dio5* exhibited a tissue-specific expression, with transcripts detected mainly in tubers and bulbils but hardly in the other tissues (Figure 2D), further strengthening the importance of *Da-dio5* in yam tuber development. To identify the cellular compartment in which Da-dio5 functions, the Da-dio5 protein fused with a green fluorescent protein (GFP) was transiently expressed in rice protoplasts. As shown in Figure 3, Da-dio5-GFP was localized to the vacuole and cytosol of transformed protoplasts.

### 2.4. Expression of Da-dio5 in Response to Hormones and Abiotic Stresses

**Figure 4:**
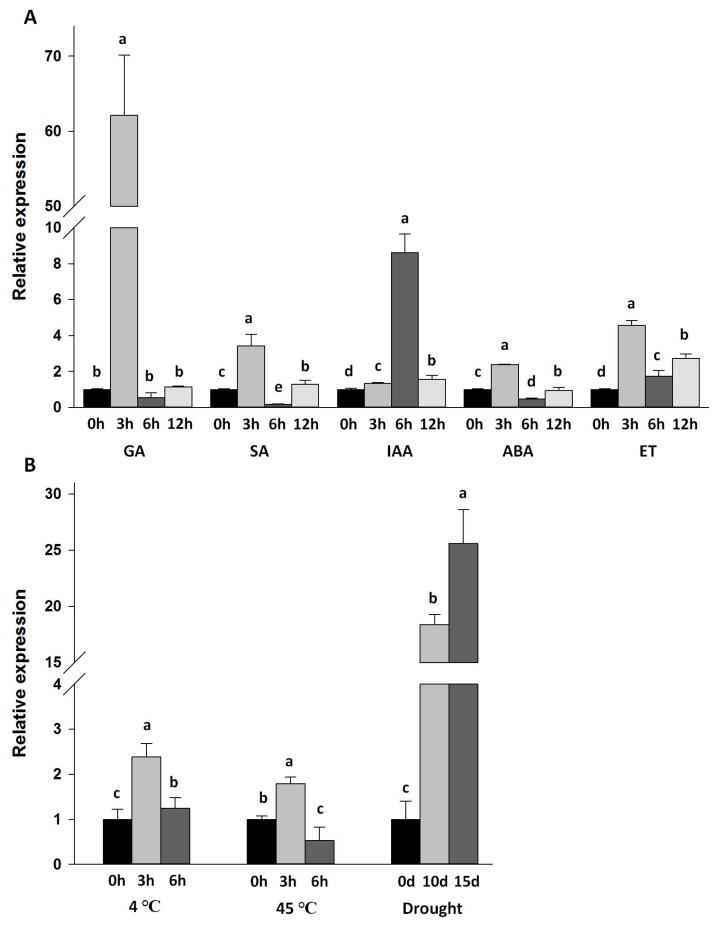
Effect of hormones and abiotic stresses on the expressions of Da-dio5 in yam leaves. qRT-PCR was conducted to determine the expressions of *Da-dio5* in leaves in response to: five hormones (GA, SA, IAA, ABA, and ET) (**A**); and three abiotic stresses (high temperature, low temperature and drought) (**B**). Values are presented as the mean ± standard error of three independent biological replicates. Different letters indicate significant differences (*p* < 0.05) according to one-way analysis of variance. GA, gibberellic acid; SA, salicylic acid; IAA, indole-3-acetic acid; ABA, abscisic acid ; ET, ethylene.

To further explore the expressional characters of *Da-dio5*, leaves of young yam plants were treated with five hormones (GA, gibberellic acid; SA, salicylic acid; IAA, indole-3-acetic acid; ABA, abscisic acid; ET, ethylene.) and three stresses (drought, 4 °C and 45 °C). As shown in Figure 4A, expressions of the *Da-dio5* in leaves were enhanced at early stages of the hormone treatments (3–6 h), but depressed in different degrees for respective hormones at later stages. The expressions of *Da-dio5* in leaves were significantly up-regulated by the drought treatment, and the two temperature stresses, although to a much lesser extent (Figure 4B).

### 2.5. Functional Characterization of the Da-dio5 Promoter

**Figure 5:**
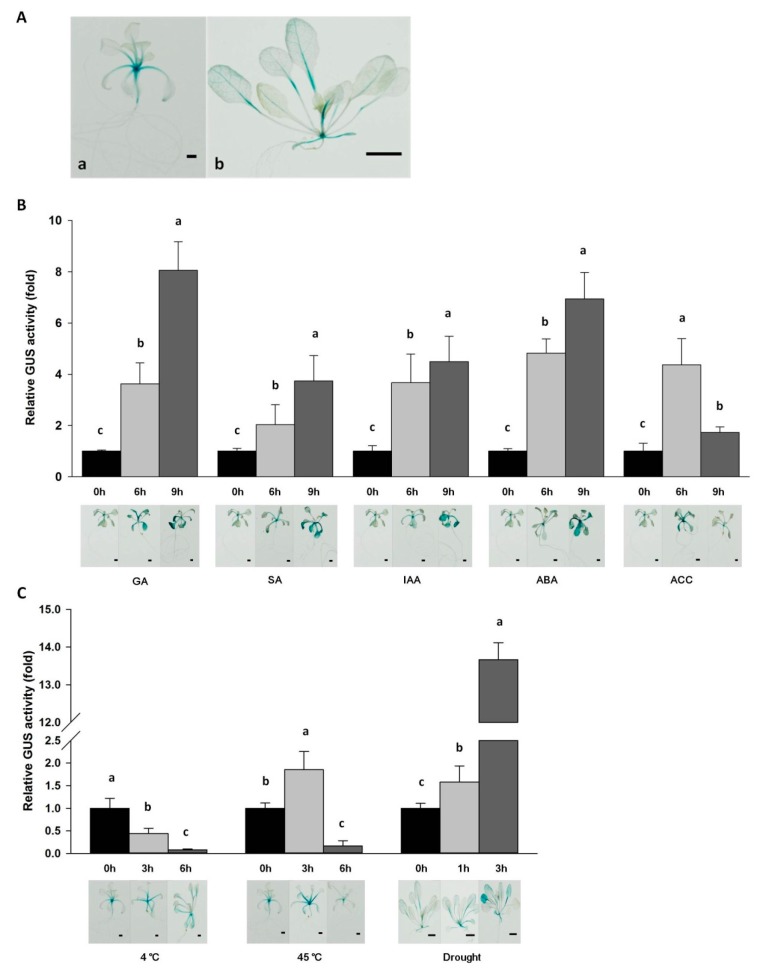
Histochemical localization and quantitative analysis of β-glucuronidase (GUS) activity in transgenic Arabidopsis plants carrying the *Da-dio5* promoter::GUS construct. (**A**) Histochemical staining of transgenic plants: (a) 10-day-old transgenic seedlings; and (b) 20-day-old transgenic seedlings. Bar = 1 mm for (a) and 1 cm for (b); (**B**) GUS activity analysis in 10-day-old transgenic seedlings after GA, SA, IAA, ABA, and ACC treatments. Bar = 1 mm; (**C**) GUS activity analysis in transgenic seedlings after low-temperature (4 °C), high-temperature (45 °C), and drought treatments. Ten-day-old transgenic seedlings were used for 4 °C and 45 °C treatments, bar = 1 mm; 20-day-old transgenic seedlings were used for drought treatment, bar = 1 cm. Values are presented as the mean ± standard error of three independent biological replicates. Different letters indicate significant differences (*p* < 0.05) according to one-way analysis of variance.

To characterize the spatiotemporal expression patterns of *Da-dio5*, a 2617-bp putative promoter fragment upstream of *Da-dio5* was cloned using a PCR-based genome walking technique. Multiple types of putative *cis*-acting elements were identified in the *Da-dio5* promoter (Table 2). The *Da-dio5* promoter contained a GCN4_motif and a Skn-1_motif, which are associated with endosperm expression. The promoter also harbored putative regulatory elements responsible for responses to environmental stresses and hormone signals, such as low-temperature and heat-stress responsive elements, drought-inducible elements, defense-responsive elements, and elements responsive to various hormones (ET, SA, GA, ABA, and IAA). For a precise analysis of the expression patterns of the *Da-dio5* promoter, we transformed the *Da-dio5* promoter::GUS chimeric construct into *A. thaliana*. In transgenic *Arabidopsis* seedlings at 10 days after germination (DAG), GUS staining was predominantly observed in the shoot apical meristem (SAM) and hypocotyls, while the petiole was weakly stained (Figure 5A). In transgenic seedlings at 20 DAG, a similar staining pattern was observed in the SAM, petiole and vasculature, whereas no staining was observed in the hypocotyls. As expected, the blue stain was not observed in control seedlings. There was also a lack of staining in the roots, flowers and stems at any developmental stages (not shown), indicating the tissue/organ-specific activity of the *Da-dio5* promoter.

To determine whether the *cis*-acting elements as predicted in hormone and stress responses (Table 2) were functional, histochemical GUS staining and GUS activity assay were conducted in transgenic seedlings. GUS activity was significantly induced in seedlings at 9 h after the treatments of GA, IAA, SA, or ABA. In contrast, in ACC-treated seedlings, GUS gene expression increased at 6 h, and then decreased at 9 h (Figure 5B). GUS activity was enhanced by both high-temperature and drought treatments, with the latter being more striking, but inhibited markedly by low-temperature treatment (Figure 5C). The GUS staining observations correlated well the assays of GUS activity (Figure 5). These data were consistent with some of the *Da-dio5* expression patterns (Figure 4) and clearly indicated that the *Da-dio5* has crucial functions in responding to various hormone and environmental cues.

## 3. Discussion

Dioscorin, which is the major soluble protein in yam (*Dioscorea* spp.) tubers, has been isolated from various yam species [12]. In previous reports, phylogenetic analysis of dioscorins from various yam species revealed that the dioscorins were grouped into two classes (A and B) [12]. Here, we showed the presence of five dioscorin genes in *D. alata* cv. Hainan No. 56 that consists of three class A (*Da-dio1*, -*3* and -*4*) and two class B (*Da-dio2* and *-5*) isoforms. Phylogenetically, the five dioscorin genes are highly conserved, similar to their homologs in other *Dioscorea* species, implying the subjection of these genes to a similar selection pressure during evolution. Moreover, complete conservation of two cysteine residues that relate to activities of TI, DHA reductase, and MDA reductase suggest additional roles besides functions as storage proteins.

Tuber development includes three major steps: induction, initiation and tuberization. Each of these steps is accompanied by a drastic change in the expression of genes encoding a set of proteins abundantly present in tubers [21]. However, it is unclear which genes are required for tuber development. In contrast to the established roles of potato (patatin) and sweet potato (sporamin) storage proteins in tuber development [21,22,23,24,25,26], dioscorins have been studied mainly for their biological activities and pharmaceutical potentials [5]. In this study, five dioscorin genes exhibited similar expression patterns during yam sprouting, regrowth and tuberization (Figure 2). The dioscorin gene expression levels gradually decreased until they were undetectable with the process of tuber germination, sprouting and regrowth (Figure 2A). Tuber growth is reported to be accompanied by protein (dioscorin) depletion along with sugar mobilization, which is controlled by the redox status of the tubers [27]. Increasing evidences have shown that dioscorins possess CA, DHA reductase and MDA reductase activities that are important for control of redox status in *D. alata* L. tubers [28]. Thus, these results together conclude that dioscorins function as the major storage protein to support tuber germination, and provide nutrients for the growth of new plants from reproductive organs. Additionally, all five dioscorin isoforms were detected at 120 DAP in accordance with tuber formation, and were highly abundant in maturing tubers (at 210 days), with Da-dio5 being the most prominent isoform (Figure 2C). Previous studies showed that the process of tuberization is accompanied by increased oxidative stress, evidenced by elevated levels of H_2_O_2_ and reactive nitrogen species. Therefore, it is suggested dioscorins have an role in controlling redox status that is also important for the process of tuberizaion [28]. Further study revealed the expressional predominance of *Da-dio*5 in the types of reproductive organs: tubers and bulbils (Figure 2D). Together, these results suggest the importance of dioscorins in both vegetative growth and reproductive development in yam plants.

The findings in sweet potato illustrate the precise roles of the phytohormone and signaling pathways in regulating expressions of tuber storage proteins (sporamin) upon abiotic stresses [29]. In this study, the expressions of *Da-dio*5 in yam leaves were up-regulated by the treatments of five phytohormones (GA, SA, IAA, ABA, and ET), and three abiotic stresses (high-temperature, low-temperature and drought) (Figure 4A,B). Thus, the induced expression of *Da-dio*5 revealed its involvement in adaptation to changes of environmental cues and stresses. Furthermore, under field conditions, induction of tuberization is controlled by a number of environmental conditions such as photo-period, temperature and nitrogen supply, via the change of endogenous phytohormone levels. Considering the expressional predominance of *Da-dio*5 in yam tubers, and the coincidence of increased dioscorin expression with yam tuberization, characterization of the *Da-dio*5 promoter will be beneficial to understanding the regulatory mechanisms of dioscorin genes in relation to their physiological functions in yam plants. Transgenic *Arabidopsis* seedlings harboring the *Da-dio*5 promoter-GUS construct showed GUS staining mainly in the SAM and hypocotyls at 10 DAG, but absent in the hypocotyls at 20 DAG (Figure 5A), indicating a strict spatial/temporal expression pattern. This is consistent with the findings that the underground stem tubers of yam are derived from swollen hypocotyls [1]. In addition, both GUS activity assay and GUS staining showed that the *Da-dio5* promoter was also induced by multiple phytohormones (GA, SA, IAA, ABA, and ACC), high-temperature and drought treatments, but inhibited by low-temperature treatment (Figure 5B,C), suggesting a multifaceted response of *Da-dio5* to changeable environmental cues. These responses could be backed up by the relevant *cis-*elements predicted in the *Da-dio5* promoter (Table 2), suggesting our observations were similar to those of tuber storage protein homologs in potato and sweet potato [30,31].

## 4. Materials and Methods

### 4.1. Plant Materials

*D. alata* cv. Hainan No. 56 plants were cultivated in the experimental plantation of the Agricultural College at Hainan University in Danzhou, Hainan, China. To clone the dioscorin genes and analyze their tissue expressions, we harvested the leaves, stems, roots, and tubers at 180 days after planting (DAP), and the male flowers at 210 DAP and the bulbils 240 DAP. To analyze gene expressions in mother tubers during yam sprouting and regrowth, we collected the mother tubers at 15, 30, 45, 60, 75, and 90 DAP after planting. To examine gene expressions during the tuber development, we collected the new tubers at the stage of tuber formation (120 DAP), the early stage of rapidly bulking (150 DAP), the rapidly bulking stage (180 DAP), the maturing stage of tubers (210 DAP), and the harvesting stage (240 DAP) (Figure 2B) [32]. We collected samples from three plants (biological replicates) for analyses. All plant materials collected were immediately frozen in liquid nitrogen and stored at −80 °C prior to RNA extraction.

*D. alata* L. plants (*n* = 3) with 6–8 fully expanded leaves were grown in a growth chamber at 28 °C under a 16-h light: 8-h dark photoperiod. *Arabidopsis thaliana* plants (ecotype Columbia) were grown in a growth chamber at 22 °C under a 16-h light: 8-h dark photoperiod.

### 4.2. Hormone and Abiotic Stress Treatments

For hormone treatments, *D. alata* L. plants (*n* = 3) with 6–8 fully expanded leaves were sprayed with 100 μM gibberellic acid (GA), 100 μM indole-3-acetic acid (IAA), 100 μM abscisic acid (ABA), 0.05% ethephon (an ethylene-releasing compound) (ET) and 100 μM salicylic acid (SA) for 12 h [29]; 10-day-old transgenic Arabidopsis seedlings were transferred to filter papers soaked with 100 μM GA, 100 μM IAA, 100 μM ABA, 100 μM 1-aminocyclopropanecarboxylic acid (ACC) and 100 μM SA for 9 h, and with double-distilled water as the control [33]. For low-temperature and high-temperature stress treatments, *D. alata* L. plants (*n* = 3) with 6–8 fully expanded leaves and 10-day-old transgenic *Arabidopsis* seedlings were transferred to filter papers saturated with MS medium and incubated at 4 °C and 45 °C for 6 h [34]. For drought stress treatment, *D. alata* L. plants (*n* = 3) with 6–8 fully expanded leaves were dehydrated for 15 d and 20-day-old transgenic *Arabidopsis* seedlings were dehydrated on filter paper at 60% humidity for 3 h [33]. Whole transgenic *Arabidopsis* seedlings were collected after treatments and assayed for GUS activity or histochemically analyzed by GUS staining as described [35,36]. The experiments were repeated three times, and with three technical replicates (10–15 seedlings per replicate).

### 4.3. Isolation of Dioscorin Genes 

A *D. alata* cv. Hainan No. 56 transcriptome database that includes a total of 52,866 unigenes was obtained by RNA-sequencing and de novo assembly using the Illumina HiSeq 2000 platform (data, unpublished). The sequences of dioscorin genes from other yam species were downloaded from the National Center for Biotechnology Information (NCBI) database and used as query sequences for basic local alignment search tool (BLAST) analysis of our transcriptome database. We obtained five *D. alata* dioscorin (*Da-dio*) target sequences with complete open reading frames. Subsequently, specific primer pairs were designed to amplify respective *Da-dio* cDNA sequences (Table S2).

### 4.4. Multiple Sequence Alignments and Bioinformatic Analysis

We deposited the obtained sequences to the NCBI database for basic local alignment search tool (BLAST) searches, following the online comparative and bioinformatic analyses of the *Da-dio* genes (Available online: http://www.ncbi.nlm.nih.gov). The amino acid sequences of the five Da-dios were aligned with homologous sequences downloaded from NCBI using the DNAMAN (Lynnon Biosoft, Quebec, QC, Canada) program. Additionally, the neighbor-joining method of the MEGA6 program was used to construct a phylogenetic tree. The molecular weights and theoretical pI (isoelectric point) values of the dioscorins were calculated with the ProtParam online tool (Available online: http://www.expasy.ch/tools/protparam.html). Potential conserved domains and signal peptides were analyzed using the SMART program (Available online: http://smart.embl-heidelberg.de/).

### 4.5. RNA Isolation and cDNA Synthesis

Total RNA was isolated from frozen plant samples using the RNAplant Plus reagent (BioTeke, Beijing, China). First-strand cDNA was synthesized using the RevertAid™ First Strand cDNA Synthesis Kit (Fermentas, Burlington, ON, Canada).

### 4.6. qRT-PCR

qRT-PCR assay was used to analyze Da-dio gene expressions, with β-actin and α-tubulin-1 as internal controls for data normalization. The efficiency of each primer pair was determined to be 1.946–2.001. The qRT-PCR was conducted using SYBR^®^ Premix Ex Taq™ II (Takara, Dalian, China) and the LightCycler 2.0 system (Roche Diagnostics, Basel, Switzerland). We used the LightCycler Relative Quantification Software 4.05 (Roche Diagnostics, Mannheim, Germany) to visualize and analyze the data, including the quantification cycle values, PCR efficiencies, and correlation coefficients. We calculated relative expression level fold changes using previously described methods [37].

### 4.7. Subcelluar Localization of Da-dio5

For subcellular localization analysis, the *Da-dio5* cDNA was amplified using the specific primer pairs of Da-dio5-GF and Da-dio5-GR (Table S3), and then cloned into the binary vector pCAMBIA-1300 at the restriction sites of *Sal* I and *Kpn* I to make the 35S::Da-dio5-GFP fusion construct. Fusion construct was transformed into *Oryza sativa* mesophyll protoplasts according to a previous study [38]. The images were acquired with laser scanning confocal microscope (Olympus FV1000, Tokyo, Japan). The excitation and emission wavelengths were 480 nm and 510 nm for GFP, and 650 nm and 750 nm for chloroplast, respectively.

### 4.8. Promoter Cloning and Analysis

The 5′-flanking region of the *Da-dio5* gene was isolated using the GenomeWalker Universal Kit (Takara, Dalian, China). The GenomeWalker libraries were constructed using genomic DNA extracted from yam leaves using the cetyltrimethylammonium bromide extraction method as previously described [39]. Primary and nested PCRs were conducted using adapter primers provided by the kit and three gene-specific antisense primers (Table S3). The PCR products were ligated into the pMD18-T vector (Takara, Dalian, China), and then sequenced at Invitrogen, China. Regulatory elements in the promoter were analyzed using the Plantcare online program (Available online: http://bioinformatics.psb.ugent.be/webtools/plantcare/html/) [40].

### 4.9. Construction of the Da-dio5 Promoter-GUS Fusion and A. thaliana Transformation

To construct the *Da-dio5* promoter-GUS fusion, the 1500-bp *Da-dio5* promoter fragment was subcloned into the *Sac* I–*Nco* I site of pCAMBIA1301. The construct was transferred into *Agrobacterium tumefaciens* EHA105 cells using the liquid nitrogen freeze–thaw method [35] for the subsequent transformation of Arabidopsis plants using the floral dip method [41]. Homozygous plants were selected from the T_2_ progenies and confirmed in the T_3_ generation according to hygromycin (50 μg/mL) resistance.

### 4.10. Histochemical Localization and Quantitative Analysis of GUS Activity

Histochemical localization of GUS activity in the homozygous transgenic plants was conducted as described [36]. Quantitative analysis of GUS activity in transgenic seedlings was as described by Lü et al. [41]. Each assay was repeated three times, with three technical replicates each time.

## Figures and Tables

**Table 1:** Basic information for all five *Dioscorea alata* dioscorin genes.

Gene Name	Accession Number	ORF Length (bp)	Predicted Protein		
			Length (aa)	MW (kDa)	pI
*Da-dio1*	KX237676	822	273	30.89	7.02
*Da-dio2*	KX237677	822	273	31.29	6.17
*Da-dio3*	KX237678	822	273	30.97	7.02
*Da-dio4*	KX237679	831	276	31.31	6.25
*Da-dio5*	KX237680	819	272	31.01	6.45

**Table 2:** Putative *cis*-elements of *Da-dio5* promoter predicted by the Plantcare online program.

Name	Sequence	Function
GCN4_motif	CAAGCCA	involved in endosperm expression element
Skn-1_motif	GTCAT	required for endosperm expression regulatory element
ABRE	GCCACGTACA	abscisic acid-responsiveness element
AuxRR-core	GGTCCAT	auxin -responsiveness element
ERE	ATTTCAAA	ethylene-responsive element
GARE- motif	TCTGTTG	gibberellin-responsive element
P- box	CCTTTTG	gibberellin-responsive element
TCA-element	CCATCTTTTT	salicylic acid-responsiveness element
HSE	AAAAAATTTC	heat stress-responsiveness element
LTR	CCGAAA	low temperature-responsiveness
MBS	CAACTG(CGGTCA)	MYB binding site involved in drought-inducibility
TC-rich repeats	ATTTTCTTCA	defense and stress responsiveness element

## Supplementary Materials

Supplementary materials can be found at www.mdpi.com/1422-0067/18/7/1579/s1.

## Abbreviations

ORF	Open reading frame
aa	Amino acids
MW	Molecular weights
bp	Base pair
BLAST	Basic local alignment search tool
TI	Trypsin inhibitor
CA	Carbonic anhydrase
DHA	Dehydroascorbate
MDA	Monodehydroascorbate
pI	Isoelectric point
qRT-PCR	Quantitative real-time RT-PCR
DAP	Days after planting
SAM	Shoot apical meristem
DAG	Days after germination
WT	Wild-type
ET	Ethylene
MeJA	Methyl jasmonate
ABA	Abscisic acid
IAA	Indole-3-acetic acid
SA	Salicylic acid
GA	Gibberellic acid
ACC	1-Aminocyclopropanecarboxylic acid
GUS	β-Glucuronidase
GFP	Green fluorescent protein

## References

[1] Shewry P.R. (2003). Tuber storage proteins. Ann. Bot..

[2] Wanasundera J.P., Ravindran G. (1994). Nutritional assessment of yam (*Dioscorea alata*) tubers. Plant Foods Hum. Nutr..

[3] Bhandari M.R., Kasai T., Kawabata J. (2003). Nutritional evaluation of wild yam (*Dioscorea* spp.) tubers of Nepal. Food Chem..

[4] Bhandari M.R., Kawabata J. (2004). Organic acid, phenolic content and antioxidant activity of wild yam (*Dioscorea* spp.) tubers of Nepal. Food Chem..

[5] Nagai T., Nagashima T. (2006). Functional properties of dioscorin, a soluble viscous protein from Japanese yam (*Dioscorea opposita* thunb.) tuber mucilage Tororo. Z. Naturforsch. C.

[6] Fu S.L., Hsu Y.H., Lee P.Y., Hou W.C., Hung L.C., Lin C.H., Chen C.M., Huang Y.J. (2006). Dioscorin isolated from *Dioscorea alata* activates TLR4-signaling pathways and induces cytokine expression in macrophages. Biochem. Biophys. Res. Commun..

[7] Fu L.S., Ko Y.H., Lin K.W., Hsu J.Y., Chu J.J., Chi C.S. (2009). Dioscorin protects tight junction protein expression in A549 human airway epithelium cells from dust mite damage. J. Microbiol. Immunol. Infect..

[8] Araghiniknam M., Chung S., Nelson-White T., Eskelson C., Watson R.R. (1996). Antioxidant activity of dioscorea and dehydroepiandrosterone (DHEA) in older humans. Life Sci..

[9] Akanbi C.T., Gureje P.O., Adeyemi I.A. (1996). Effect of heat-moisture pre-treatment on physical characteristics of dehydrated yam. J. Food Eng..

[10] Omonigho S.E., Ikenebomeh M.J. (2000). Effect of temperature treatment on the chemical composition of pounded white yam during storage. Food Chem..

[11] Harvey P.J., Boulter B. (1983). Isolation and characterization of the storage protein of yam tubers (*Dioscorea rotundata*). Phytochemistry.

[12] Tsai W.Y., Jheng Y.J., Chen K.H., Lin K.W., Ho Y.P., Yang C.C., Lin K.C. (2013). Molecular cloning, structural analysis and mass spectrometric identification of native dioscorins of various yam species. J. Sci. Food Agric..

[13] Hou W.C., Chen H.J., Lin Y.H. (2000). Dioscorins from different Dioscorea species all exhibit both carbonic anhydrase and trypsin inhibitor activities. Bot. Bull. Acad. Sin..

[14] Gaidamashvili M., Ohizumi Y., Iijima S., Takayama T., Ogawa T., Muramoto K. (2004). Characterization of the yam tuber storage proteins from *Dioscorea batatas* exhibiting unique lectin activities. J. Biol. Chem..

[15] Hou W.C., Chen H.J., Lin Y.H. (1999). Dioscorins, the major tuber storage proteins of yam (*Dioscorea batatas* Decne), with dehydroascorbate reductase and monodehydroascorbate reductase activities. Plant Sci..

[16] Hou W.C., Liu J.S., Chen H.J., Chen T.E., Chang C.F., Lin Y.H. (1999). Dioscorin, the major tuber storage protein of yam (*Dioscorea batatas* decne) with carbonic anhydrase and trypsin inhibitor activities. J. Agric. Food Chem..

[17] Hou W.C., Lee M.H., Chen H.J., Liang W.L., Han C.H., Liu Y.W., Lin Y.H. (2001). Antioxidant activities of dioscorin, the storage protein of yam (*Dioscorea batatas* Decne) tuber. J. Agric. Food Chem..

[18] Hsu F.L., Lin Y.H., Lee M.H., Lin C.L., Hou W.C. (2002). Both dioscorin, the tuber storage protein of yam (*Dioscorea alata* cv. Tainong No. 1), and its peptic hydrolysates exhibited angiotensin converting enzyme inhibitory activities. J. Agric. Food Chem..

[19] Yeh K.W., Chen J.C., Lin M.I., Chen Y.M., Lin C.Y. (1997). Functional activity of sporamin from sweet potato (*Ipomoea batatas* Lam.): A tuber storage protein with trypsin inhibitory activity. Plant Mol. Biol..

[20] Racusen D. (1984). Lipid acyl hydrolase of patatin. Can. J. Bot..

[21] Hendriks T., Vreugdenhil D., Stiekema W.J. (1991). Patatin and four serine proteinase inhibitor genes are differentially expressed during potato tuber development. Plant Mol. Biol..

[22] Park W., Hannapel D., Mignery G., Pikaard C. (1985). Molecular approaches to the study of the major tuber proteins. Potato Physiol..

[23] Maeshima M., Sasaki T., Asahi T. (1985). Characterization of major proteins in sweet potato tuberous roots. Phytochemistry.

[24] Hattori T., Nakagawa T., Maeshima M., Nakamura K., Asahi T. (1985). Molecular cloning and nucleotide sequence of cDNA for sporamin, the major soluble protein of sweet potato tuberous roots. Plant Mol. Biol..

[25] Hannapel D.J. (1991). Characterization of the early events of potato tuber development. Physiol. Plant.

[26] Paiva E., Lister R.M., Park W.D. (1983). Induction and accumulation of major tuber proteins of potato in stems and petioles. Plant Physiol..

[27] Sharma S., Gupta R., Deswal R. (2017). *Dioscorea alata* tuber proteome analysis shows over thirty dioscorin isoforms and novel tuber proteins. Plant Physiol. Biochem..

[28] Sharma S., Sehrawat A., Deswal R. (2016). Asada-Halliwell pathway maintains redox status in *Dioscorea alata* tuber which helps in germination. Plant Sci..

[29] Rajendran S., Lin I.W., Chen M.J., Chen C.Y., Yeh K.W. (2014). Differential activation of sporamin expression in response to abiotic mechanical wounding and biotic herbivore attack in the sweet potato. BMC Plant Biol..

[30] Twell D., Ooms G. (1987). The 5′ flanking DNA of a patatin gene directs tuber specific expression of a chimaeric gene in potato. Plant Mol. Biol..

[31] Rosahl S., Schell J., Willmitzer L. (1987). Expression of a tuber-specific storage protein in transgenic tobacco plants: Demonstration of an esterase activity. EMBO J..

[32] Zhou M.S., Wang P.L., Xiang X., Wei H.B., Li Z.L., Li R.Y., Fang X.F., Cao S.J. (2009). Cloning and molecular characteristics of ANS gene and its correlations with anthocyan in accumulation in yam. Acta Hortic. Sin..

[33] Wang S.J., Lan Y.C., Chen S.F., Chen Y.M., Yeh K.W. (2002). Wound-response regulation of the sweet potato sporamin gene promoter region. Plant Mol. Biol..

[34] Eun C.H., Kim S.U., Kim I.J. (2015). The promoter from the Citrus unshiucarotenoid isomerase gene directs differential GUS expression in transgenic Arabidopsis. Mol. Breed..

[35] Lü S., Gu H., Yuan X., Wang X., Wu A.M., Qu L., Liu J.Y. (2007). The GUS reporter-aided analysis of the promoter activities of a rice metallothionein gene reveals different regulatory regions responsible for tissue-specific and inducible expression in transgenic Arabidopsis. Transgenic Res..

[36] Jefferson R.A., Kavanagh T.A., Bevan M.W. (1987). GUS fusions: β-Glucuronidase as a sensitive and versatile gene fusion marker in higher plants. EMBO J..

[37] Vandesompele J., de Preter K., Pattyn F., Poppe B., van Roy N., de Paepe A., Speleman F. (2002). Accurate normalization of real-time quantitative RT-PCR data by geometric averaging of multiple internal control genes. Genome Biol..

[38] Sheen J. (2001). Signal transduction in maize and Arabidopsis mesophyll protoplasts. Plant Physiol..

[39] Randhawa G.J., Singh M., Chhabra R. (2013). DNA-based diagnostics for genetically modified cotton: Decaplex PCR assay to differentiate MON531 and MON15985 Bt cotton events. Methods Mol. Biol..

[40] Lescot M., Dehais P., Thijs G., Marchal K., Moreau Y., van de Peer Y., Rouze P., Rombauts S. (2002). PlantCARE, a database of plant *cis*-acting regulatory elements and a portal to tools for in silico analysis of promoter sequences. Nucleic Acids Res..

[41] Clough S.J., Bent A.F. (1998). Floral dip: A simplified method for Agrobacterium-mediated transformation of Arabidopsis thaliana. Plant J..

