# Supplementary material for: Characterization of the Dioscorin Gene Family in Dioscorea alata Reveals a Role in Tuber Development and Environmental Response
Source: Int J Mol Sci. 2017 Jul 20;18(7):1579. doi: 10.3390/ijms18071579 (PMC5536067; doi:10.3390/ijms18071579)
Supplement: Supplementary file 1 [file ijms-18-01579-s001.pdf]

Supplementary tables:

Table S1. Amino acid sequence identities among five *Da-dios* genes

	Da-dio1	Da-dio2	Da-dio3	Da-dio4	Da-dio5	Da-dioA1	Da-dioA2	Da-dioA3	Da-dioB1
Da-dio1	100	68	96	94	68	91	92	86	67
Da-dio2		100	68	68	91	69	71	62	97
Da-dio3			100	97	68	94	95	89	66
Da-dio4				100	68	93	93	87	66
Da-dio5					100	68	71	62	90
Da-dioA1						100	91	87	68
Da-dioA2							100	84	71
Da-dioA3								100	61
Da-dioB1									100

Table S2. Primer sequences used in cDNA amplification and qPCR analysis

**Primer code**	**Primer sequence**	**Usage**
Da-dio1-F	5′-TAACAATCATGAGTTCATCCACCC-3′	cDNA amplification
Da-dio1-R	5′- TTATACATAGGATGATAGTACATGCAGA-3′	
Da-dio2-F	5′-TAACAATCATGAGTTCATCCACCC-3′	
Da-dio2-R	5′-CCACTCTTTTTATTTAACCATCACC-3′	
Da-dio3-F	5′-ATTATGGCCGGGGACTC-3′	
Da-dio3-R	5′-TTACGTGCTTCCTTAATTATCATA-3′	
Da-dio4-F	5′-ATTATGGCCGGGGACTC-3′	
Da-dio4-R	5′-TTACGTGCTTCCTTAATTATCATA-3′	
Da-dio5-F	5′- CCGGGGACTCCAATCTAAGC-3′	
Da-dio5-R	5′-GCTACATGCAGAGTGGAGCA-3′	

Da-dio1-Q-F	5′-AGTGCTGACCCATTCCTCTCG-3′	qPCR analysis (target genes)
Da-dio1-Q-R	5′-TCCCAAGTATCTAAAATAAGCAGAGTCA-3′	
Da-dio2-Q-F	5′-TCCCTCCTCTTCTCTTGCTTTT-3′	
Da-dio2-Q-R	5′-CCGTATCACTCTATCATCACACAAAT-3′	
Da-dio3-Q-F	5′-GAAATAAATGCAGGAGTTGTGGATA-3′	
Da-dio3-Q-R	5′-TCATTCACTGCCTGCTTCAACA-3′	
Da-dio4-Q-F	5′-AGCAATAGCCAGAAGAATGAAATC-3′	
Da-dio4-Q-R	5′-TGCAACCTTCCTCATGACGG-3′	
Da-dio5-Q-F	5′-CCCTCCTCTTCTCTTGCTTATCAT-3′	
Da-dio5-Q-R	5′-CGTATCACTCTATCATCACACAAACA-3′	
β-Actin-F	5′-GGGTTACTCATTCACCACCACAG-3′	qPCR analysis (reference genes)
β-Actin-R	5′-GGTAACTCATAACTCTTCTCAACAGCA-3′	
Tubulin-F	5′-CTTGGCTTTACCATCTATCCTTCC-3′	
Tubulin-R	5′-AGTGTGTTCAAGTAAGGAATGGGT-3′	

Table S3. Primer sequences used for amplification of *Da-dio5* promoter and construction of binary vectors

**Primer code**	**Primer sequence**	**Usage**
SP1	5′-TGAAAATGAATTCGCTTGAGTTGGTACTG-3′	Promoter amplification
SP2	5′-GAGTCCGTATCACTCTATCATCACACAAACA-3′	
SP3	5′-ACACAAACAAATGGGTGACTGCTCCT-3′	
Da-dio5QI-F	5′-GCGGAGCTCAGATCCTTTTTCTGGGCTGGT-3′	*Da-dio5*::*GUS* transformation
Da-dio5QI-R	5′-CATGGGTCTCCCATGGTTGTCTTTCTTAATTTTTTGC-3′	
Da-dio5-GF	5′-ACAGTCGACATGAGTTCATCCACCCTTTTCCAT-3′	Subcelluar localization
Da-dio5-GR	5′-ACAGGTACCTATGGCATCATGATTCGATTTTTG-3′	

Underlined sequences indicate restriction recognition sites.
